# Utility of Metagenomic Next-Generation Sequencing for Etiological Diagnosis of Patients with Sepsis in Intensive Care Units

**DOI:** 10.1128/spectrum.00746-22

**Published:** 2022-07-21

**Authors:** Jung-Yien Chien, Chong-Jen Yu, Po-Ren Hsueh

**Affiliations:** a Department of Internal Medicine, College of Medicine, National Taiwan University Hospital, Taipei, Taiwan; b Department of Laboratory Medicine, College of Medicine, National Taiwan University Hospital, Taipei, Taiwan; c Departments of Laboratory Medicine and Internal Medicine, China Medical University Hospital, Taichung, Taiwan; d School of Medicine, China Medical University, Taichung, Taiwan; e Ph.D. Program for Aging, College of Medicine, China Medical University, Taichung, Taiwan; Institut National de Santé Publique du Québec

**Keywords:** sepsis, metagenomic next-generation sequencing, etiological diagnosis, performance, intensive care units

## Abstract

The performance of metagenomic next-generation sequencing (mNGS) was evaluated and compared with that of conventional culture testing in patients with sepsis. Prospective blood and bronchoalveolar lavage fluid (BALF) samples from 50 patients with sepsis were tested using cultures (bacterial, fungal, and viral) and mNGS of microbial DNA (blood and BALF) and RNA (BALF). mNGS had higher detection rates than blood culture (88.0% versus 26.0%, *P* < 0.001) and BALF culture (92.0% versus 76.0%, *P* = 0.054). RNA-based mNGS has increased the detection rate of several bacteria, fungi, and viruses, but not mycobacteria and Toxoplasma gondii. The number of multiple detections per specimen was higher in BALF (92.0%) than in blood (78.0%) samples, and the highest number of pathogens detected in a single specimen was 32. Among blood samples, compared to cultures, mNGS detected significantly more bacteria (*P* < 0.001), fungi (*P* = 0.012), and viruses (*P* < 0.001), whereas BALF mNGS had a higher detection rate for bacteria (*P* < 0.001) and viruses (*P* < 0.001). The percentage of mNGS-positive samples was significantly higher than that of culture-positive samples for several Gram-negative bacteria, some Gram-positive bacteria, and viruses, but not fungi. Mycobacteria had a higher detection rate by culture than by mNGS, but the difference was not significant due to the small sample size. The positive and negative agreements with 95% confidence intervals of mNGS and culture were 62.0% (50.4 to 72.7) and 96.8% (96.5 to 97.1), respectively. mNGS offers a sensitive diagnostic method for patients with sepsis and is promising for the detection of multipathogen infections. Clinical correlation is advised to interpret mNGS data due to the lack of unified diagnostic criteria.

**IMPORTANCE** Delays in effective antimicrobial therapy have resulted in decreased survival rates among patients with sepsis. However, current culture-based diagnostic methods have low sensitivity because of concurrent antibiotic exposure and fastidious and atypical causative organisms. Among patients with sepsis, we showed that mNGS methods had higher positive rates than culture methods, especially for bacteria, viruses, and multipathogen infections, which are difficult to culture and detect in patients treated with antibiotics. RNA-based mNGS has increased the detection rate of several bacteria, fungi, and viruses, but not mycobacteria and Toxoplasma gondii. mNGS also showed a high negative percent agreement with cultures. However, the interpretation of mNGS data should be combined with clinical data and conventional methods considering the lack of unified diagnostic criteria.

## INTRODUCTION

Sepsis remains a challenge in intensive care medicine and its incidence has increased continuously over the past few decades (1). Delays in effective antimicrobial therapy are known to result in decreased survival (2). Therefore, the correct identification of causative pathogens is crucial for optimizing antimicrobial treatment. Frequently, patients with sepsis are immunocompromised owing to cancer, hereditary syndromes, or transplantation. The causative agents of sepsis, including common and uncommon pathogens, range from viruses to bacteria, fungi, and parasites. However, current culture-based diagnostic procedures (e.g., blood and sputum cultures) often yield negative results even when bacterial or fungal infections are strongly suspected (3). This may be partially due to concurrent antibiotic treatment (4–6), prophylactic antimicrobial drugs, and fastidious or slow-growing causative organisms.

Culture-independent diagnostic techniques, such as PCR-based techniques, which do not depend on the growth of organisms in culture, may offer distinct advantages over culture-based methods (7–9). Nevertheless, most molecular methods rely on the amplification of microbial genes in culture as precursors for diagnosis (7–9). In addition, many of these methods have limited coverage for specific organisms and miss rare pathogens due to the use of mismatched primers, which decrease the sensitivity of the method (10).

Metagenomic next-generation sequencing (mNGS), known as untargeted shotgun sequencing of random DNA or RNA in samples, has recently been identified as a promising diagnostic method for the unbiased detection of pathogens at different sites. mNGS can detect pathogens causing (11) bloodstream (12), respiratory (13), central nervous system (14), ocular infections (15), and fever of unknown origin (16), especially in immunocompromised patients (17). The main advantage of mNGS is unbiased sampling, which enables the broad identification of known and unexpected pathogens as well as the discovery of new organisms (18). mNGS can provide quantitative or semiquantitative data regarding the concentration of organisms in a sample by analyzing the sequenced reads, which is useful for polymicrobial samples or in cases in which more than one pathogen is implicated in the disease process (19).

This new approach to mNGS for diagnosis might be more sensitive than traditional culture methods. However, further studies are required to precisely define the performance and clinical value of mNGS, as most previous studies were limited by their retrospective design. Therefore, this prospective study in patients with sepsis was conducted to evaluate the diagnostic utility and clinical significance of mNGS compared to standard culture-based methods.

## RESULTS

**FIG 1:**
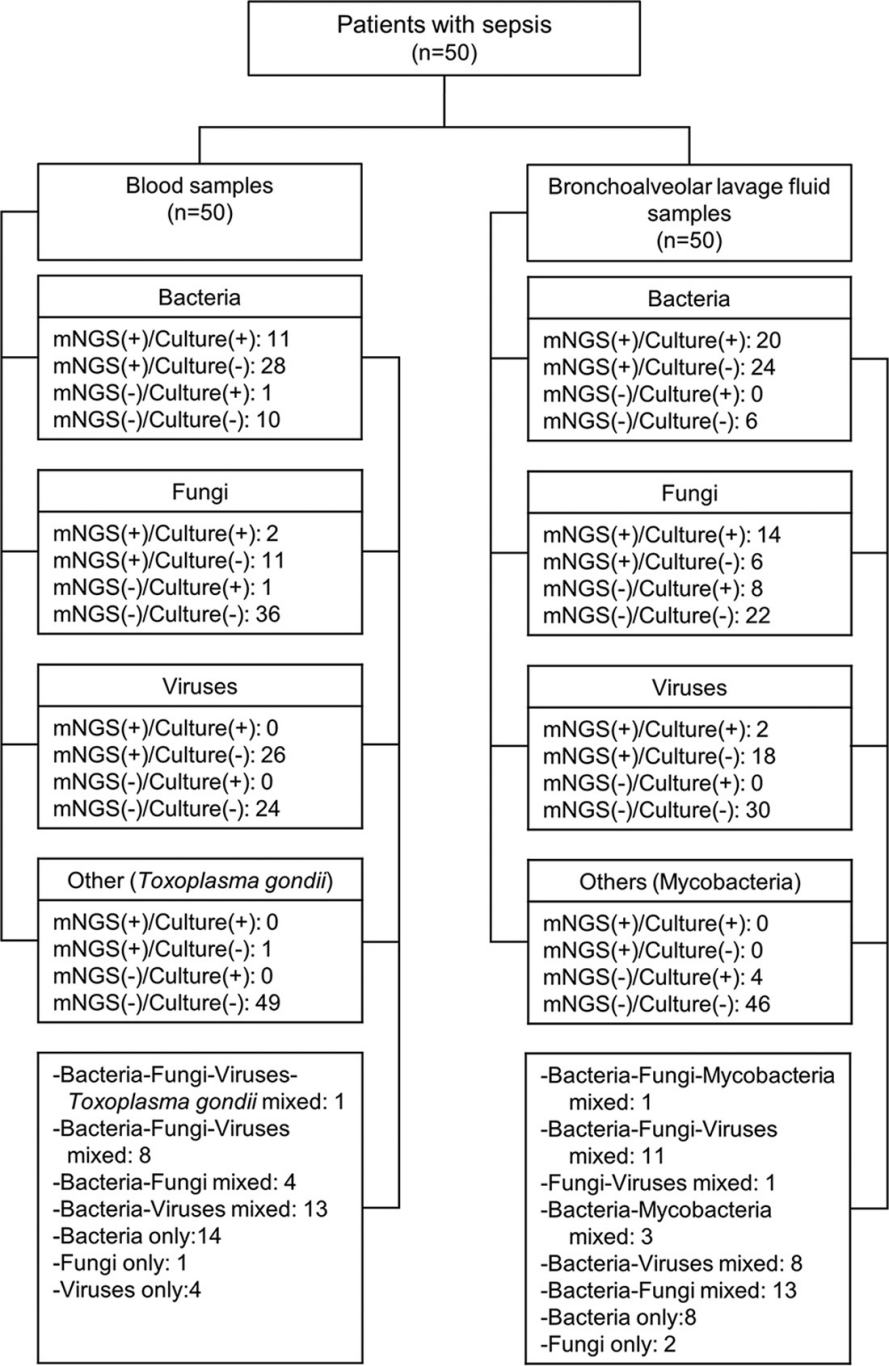
Flow chart of sample classification and comparison. Pathogens were classified as bacteria, fungi, viruses, or others.

We analyzed a total of 50 blood and 50 bronchoalveolar lavage fluid (BALF) samples from 50 adult patients with sepsis (17 women and 33 men, 41 to 101 years old) admitted to the intensive care unit (ICU) (Table 1 and Fig. 1). Of these, 33 (66.0%) had hospital-associated pneumonia, 16 (32.0%) had community-acquired pneumonia, and one (2.0%) had urosepsis. All patients experienced respiratory failure, and 24 (48.0%) died within 28 days.

**TABLE 1:** Clinical characteristics of the 50 study patients

Characteristics	Data^*a*^
Age in years	75 (41–101)
Women	17 (34.0)
APACHE II score^*b*^	23 (11–46)
SOFA score	9 (3–16)
Comorbidities	
Stroke	8 (16.0)
Dementia	3 (6.0)
Heart failure	8 (16.0)
Coronary artery disease	5 (10.0)
Valvular heart disease	3 (6.0)
Diabetes	17 (34.0)
Hypertension	29 (58.0)
Chronic kidney disease	12 (24.0)
Cirrhosis of liver	0 (0.0)
Chronic obstructive pulmonary disease	7 (14.0)
Asthma	2 (4.0)
Interstitial lung disease	3 (6.0)
Bronchiectasis	3 (6.0)
Hematological cancer	7 (14.0)
Lymphoma	3 (6.0)
Solid cancer	20 (40.0)
Autoimmune diseases	4 (8.0)
Cause of sepsis	
Hospital associated pneumonia	33 (66.0%)
Community-acquired pneumonia	16 (32.0%)
Urosepsis	1 (2.0%)
Antibiotic usage before mNGS	50 (100.0)
Hospital mortality	24 (48%)

a Data are presented as n (%) or median (range).

b SOFA, sepsis-related organ failure assessment; APACHE, acute physiology and chronic health evaluation; mNGS, metagenomic next-generation sequencing.

**FIG 2:**
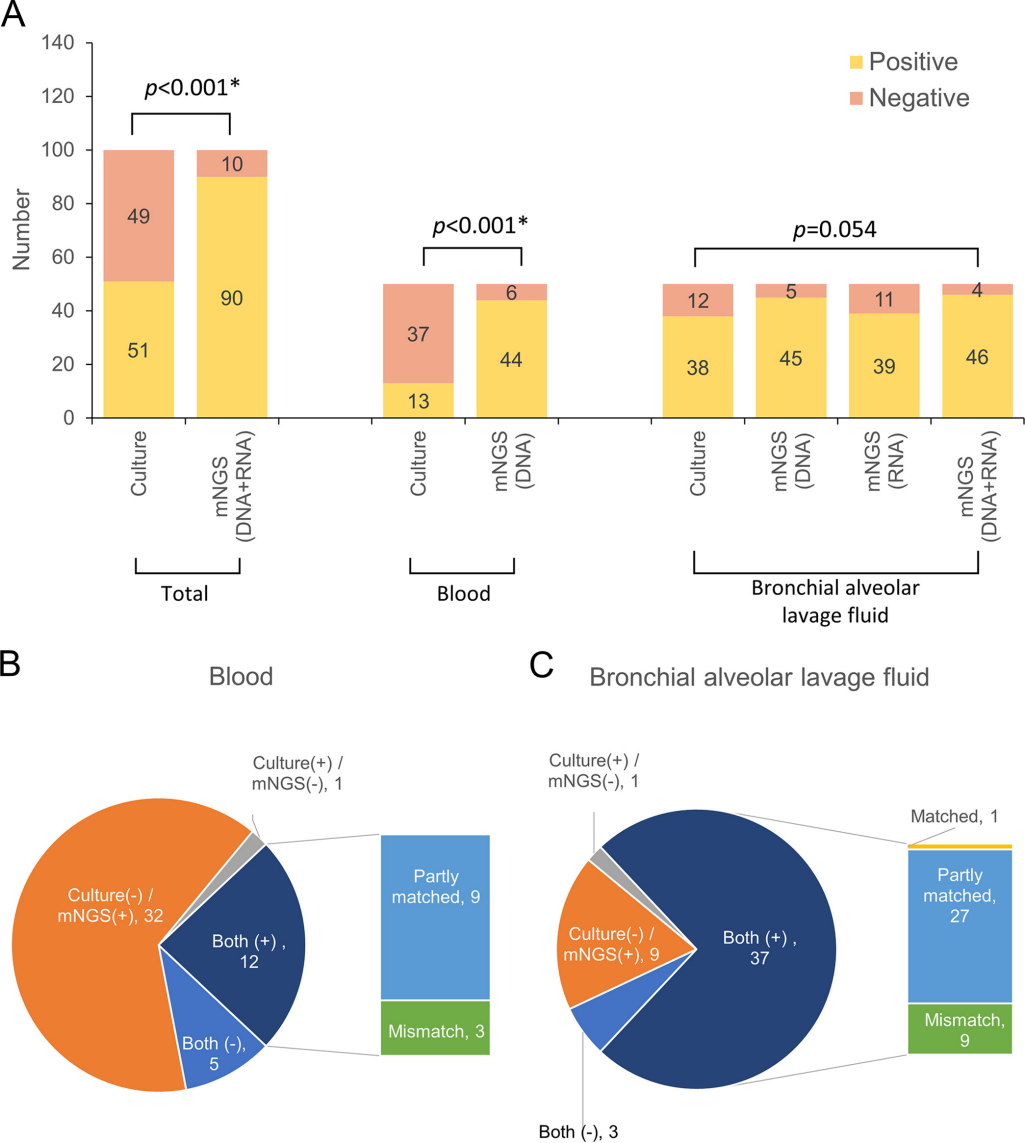
Positivity rate comparison and agreement analysis (A) between metagenomic next-generation sequencing (mNGS) and culture for detection of pathogens causing sepsis in the blood (B) and bronchoalveolar lavage fluid (BALF; C).

As shown in Fig. 2, cultures detected at least one pathogen in 51 of the 100 samples tested, in 26.0% of the blood and 76.0% of BALF samples, yielding an overall positivity rate of 51.0%. mNGS detected pathogens in 90 of the 100 specimens, in 88.0% of the blood and 92.0% of the BALF samples, yielding an overall positivity rate of 90.0%. mNGS had a higher detection rate than culture of blood (88.0% versus 26.0%, *P* < 0.001) and BALF samples (92.0% versus 76.0%, *P* = 0.054). Of the 100 samples, both mNGS and culture detected pathogens in 49 (49.0%) samples, with no pathogens detected in 8 (8.0%) samples, while pathogens in 41 (41.0%) samples were detected by mNGS alone and in 2 (2.0%) samples, they were detected by culture alone. Of the 49 samples that were positive by both mNGS and culture, the results of mNGS and culture were completely matched (all pathogens identified by mNGS were confirmed by culture) in only one (2.0%) sample and completely mismatched (all pathogens identified by mNGS could not be confirmed by culture) in 12 (24.5%). The remaining 36 (73.5%) samples were partially matched (at least one pathogen identified in one test was confirmed by the other). Fig. 2B and C show that, compared to blood samples, pathogens in a lower proportion of BALF samples were detected by mNGS alone (64.0% versus 18.0%, *P* < 0.001), and more BALF samples were partly matched (18.0% versus 54.0%, *P* < 0.001) or totally mismatched (6.0% versus 18.0%, *P* = 0.121).

**FIG 3:**
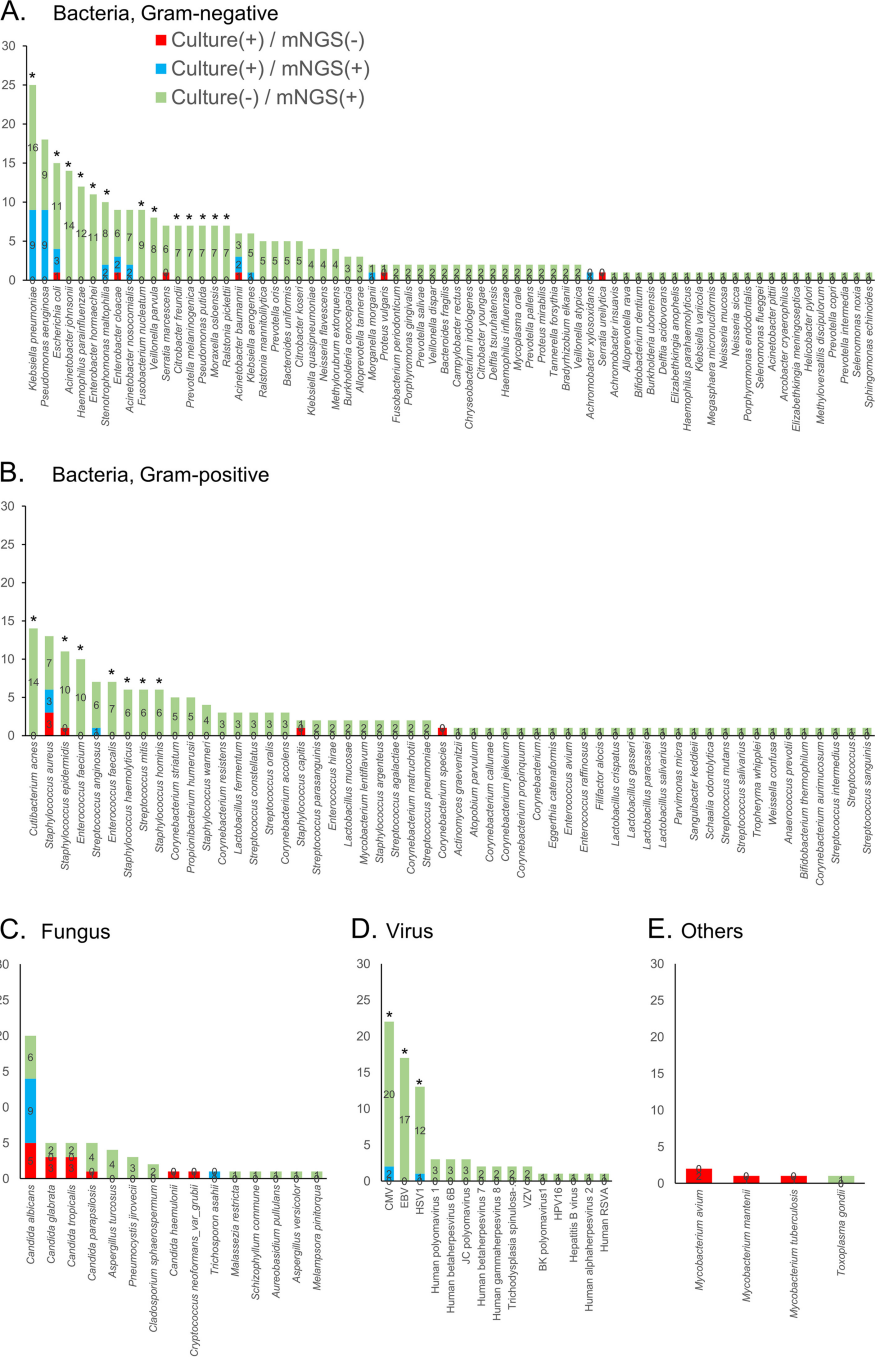
Agreement between metagenomic next-generation sequencing (mNGS) and culture in detecting different pathogens. *, *P* < 0.05; CMV, cytomegalovirus; EBV, Epstein-Barr virus; HSV1, herpes simplex virus 1; VZV, varicella-zoster virus; HPV, human papillomavirus; RSVA, respiratory syncytial virus subtype A.

Bacteria, fungi, and viruses were detected in 84 (84.0%), 42 (42.0%), and 46 (46.0%) of the samples, respectively (Table S1). A total of 158 pathogens were detected (Fig. 3), of which Klebsiella pneumoniae (25/100) was the most common, followed by cytomegalovirus (CMV) (22/100), Candida albicans (20/100), Pseudomonas aeruginosa (18/100), Epstein-Barr virus (EBV) (17/100), Escherichia coli (15/100), and Acinetobacter johnsonii (14/100). As shown in Table 2 and Table S2, additional RNA sequencing increased the detection rate of mNGS for several bacteria, fungi, and viruses, but not for mycobacteria and Toxoplasma gondii.

**TABLE 2:** Organisms detected by routine culture and metagenomic next-generation sequencing (mNGS) of blood and bronchial alveolar lavage fluid

	Blood		Bronchial alveolar lavage fluid			
Pathogen	Culture	mNGS (DNA)	Culture	mNGS (DNA)	mNGS (RNA)	mNGS (DNA/RNA)
Total	21	200	58	254	247	348
Bacteria, Gram-negative	11	99	27	139	127	187
Bacteria, Gram-positive	7	39	3	75	83	110
Fungi	3	15	21	21	13	23
Viruses	0	46	3	19	24	28
Mycobacteria	0	0	4	0	0	0
Other (Toxoplasma gondii)	0	1	0	0	0	0

**FIG 4:**
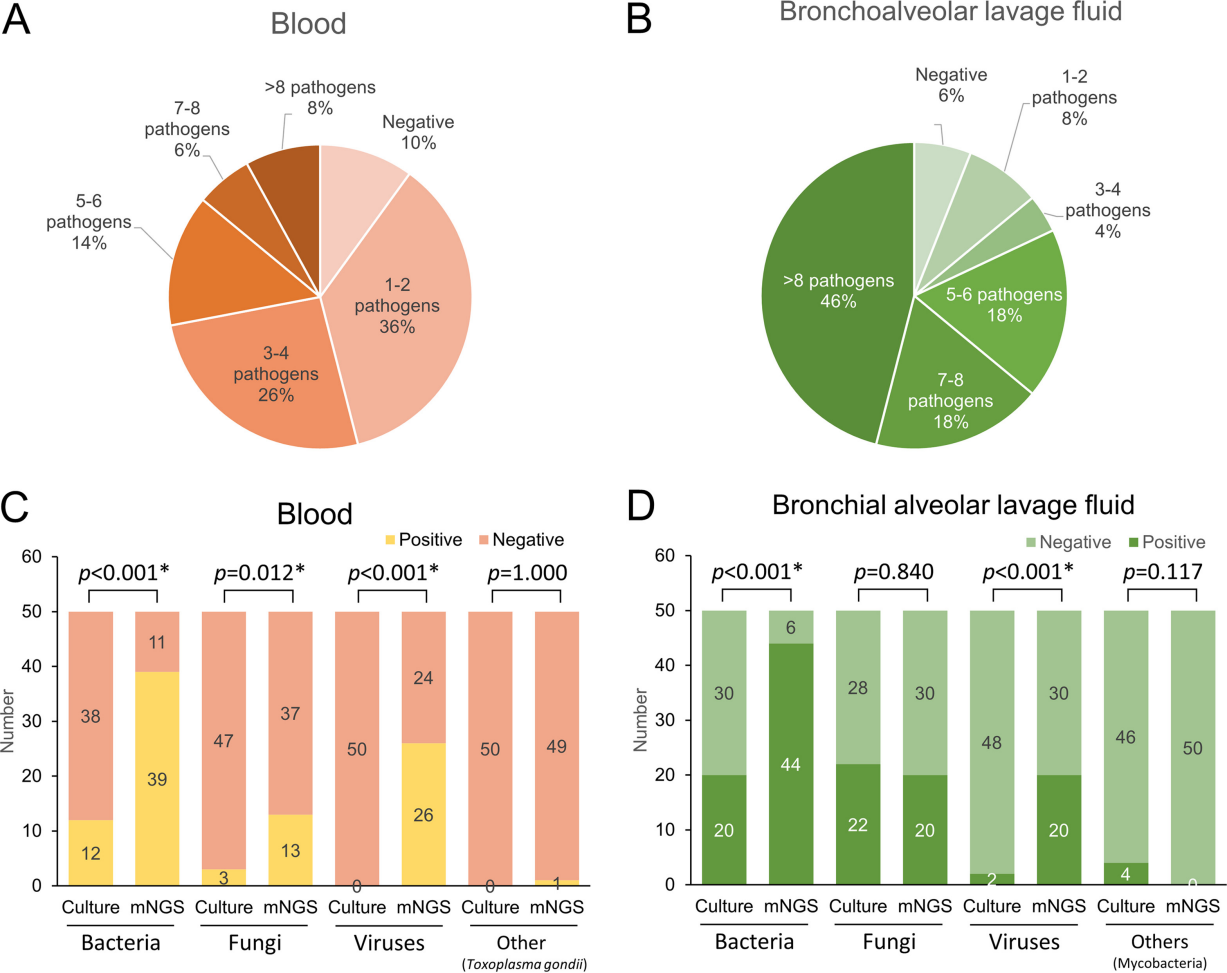
Detection of multiple pathogens per specimen (A and B), and positivity rate comparison (C and D) in blood and bronchoalveolar lavage fluid.

As shown in Fig. 4, multiple detections per specimen were higher in BALF (92.0%, 46/50) than in blood (78.0%, 39/50) samples in 85 (94.4%) of the positive samples, and the highest number of pathogens detected in a single specimen was 32. In blood samples, mNGS detected significantly more bacteria (*P* < 0.001), fungi (*P* = 0.012), and viruses (*P* < 0.001) than culture, whereas in BALF, mNGS had a higher detection rate for bacteria (*P* < 0.001) and viruses (*P* < 0.001) than conventional cultures.

Consistent identification data between culture and mNGS are shown in Table 3, Table S3, and Fig. 3. The percentage of mNGS-positive samples was significantly higher than that of culture-positive samples for several Gram-negative bacteria (K. pneumoniae, E. coli, A. johnsonii, Cutibacterium acnes, Haemophilus parainfluenzae, Enterobacter hormaechei, Stenotrophomonas maltophilia, Fusobacterium nucleatum, Veillonella parvula, Citrobacter freundii, Prevotella melaninogenica, P. putida, Moraxella osloensis, and Ralstonia pickettii), certain Gram-positive bacteria (Staphylococcus epidermidis, Enterococcus faecium, E. faecalis, S. haemolyticus, Streptococcus mitis, and S. hominis), and viruses (CMV, EBV, and herpes simplex virus 1 [HSV1]), but not fungi. Mycobacteria were detected more frequently by culture than by mNGS, but the difference was not significant due to the small sample size. The positive percent agreement (PPA) and negative percent agreement (NPA) with 95% confidence intervals of mNGS and culture were 62.0% (50.4 to 72.7) and 96.8% (96.5 to 97.1), respectively (Table 3 and Table S3). The 11 pathogens with agreement rates >80% were E. hormaechei (89.0%), S. epidermidis (89.0%), C. albicans (89.0%), E. coli (88.0%), H. parainfluenzae (88.0%), HSV1 (88.0%), A. johnsonii (86.0%), C. acnes (86.0%), K. pneumoniae (84.0%), EBV (83.0%), and CMV (80.0%).

**TABLE 3:** Agreement between routine culture and metagenomic next-generation sequencing (mNGS) detection

Pathogen	Culture(+)/mNGS(−)	Culture(+)/mNGS(+)	Culture(−)/mNGS(+)	Culture(−)/mNGS(−)	PPA (%)	NPA^*a*^ (%)
Bacteria, Gram-negative	6	32	254	6708	84.2	96.4
Bacteria, Gram-positive	6	4	145	5245	40.0	97.3
Fungi	14	10	28	1448	41.7	98.1
Viruses	0	3	71	1426	100.0	95.3
Mycobacteria	4	0	0	296	0.0	100.0
Other (Toxoplasma gondii)	0	0	1	99		99.0
All	30	49	499	15222	62.0	96.8

a NPA, negative percent agreement; PPA, positive percent agreement.

## DISCUSSION

In this study, we found that the overall pathogen detection rate using mNGS was significantly higher than that of conventional cultures, especially in blood samples. Compared to blood samples, BALF samples had a higher proportion of multiple detections per specimen. Compared to cultures, mNGS detected significantly more bacteria, fungi, and viruses in blood samples and more bacteria and viruses in BALF samples. Furthermore, mNGS had higher detection rates for several Gram-negative bacteria, some Gram-positive bacteria, and viruses, but not for fungi and mycobacteria.

The shotgun-based high-throughput mNGS method effectively detects atypical pathogens such as viruses, fungi, mycobacteria (20, 21), and *Mycoplasma* species (22). mNGS was also more sensitive in samples containing low levels of nucleic acids from microorganisms and, thus, is less affected by antimicrobial therapy (22, 23). Additionally, the rate of positive mNGS results may be constant over different time points after sepsis (24). In septic shock, Grumaz et al. (24) found a 6-fold higher positivity rate for mNGS than blood culture, and half of the positive results would lead to a change in antimicrobial therapy. Using retrospective analysis, they estimated that mNGS detection would lead to improved survival and reduce the overall use of antimicrobials, indicating the potential benefit of mNGS, even without the identification of antimicrobial resistance. In this study, culture samples were collected before empirical antibiotic use. In contrast, due to the informed process, samples for mNGS were collected 6 to 24 h after empirical antibiotic treatment for sepsis. However, mNGS still exhibited significantly higher positive rates for the detection of common pathogens associated with sepsis, such as K. pneumoniae, P. aeruginosa, and E. coli, as well as culture-negative pathogens in the blood. This indicates that mNGS can provide valuable data to assist intensive care specialists in the treatment of septic patients.

Identification of fungal infections is crucial for the treatment of sepsis (25). Camargo et al. (26) showed that mNGS can identify fungi, such as P. jirovecii and Aspergillus species, in patients receiving effective antifungal agents. A recent study by Fang et al. (27) found that, compared to culture, mNGS had a higher detection rate for Aspergillus. However, some studies have shown contrasting results (28). Compared with the culture results, we found that mNGS in blood and BALF samples had higher detection sensitivity for viruses (100%) and Gram-negative bacteria (84.2%) than for fungi (41.7%) and Gram-positive bacteria (40.0%) (Table 3 and Table S3). Compared to culture methods, mNGS had a higher detection rate for fungi in blood samples, but not in BALF samples (Fig. 4C and D).

One of the features of mNGS is its ability to simultaneously detect bacterial, fungal, and viral pathogens. Similar to the findings of previous studies (22, 25), we found that mNGS detected more concurrent pathogens in the blood (90.9% versus 38.5%, *P* < 0.001) and BALF (95.7% versus 39.5%) samples than in culture. These differences may be due to the use of antibiotics that result in bacterial death and low bacterial concentrations in samples that are difficult to culture (25). In addition, Long et al. (25) found that the predominant bacterial strains in culture could suppress the growth of other strains. Furthermore, fastidious microorganisms can go undetected under standard culture conditions (29). Viral infections are often complicated during sepsis and are critically involved in the progression and outcome of sepsis (30). Although DNA-based mNGS exhibits advantages in the simultaneous detection of bacteria, fungi, and viruses (31), DNA sequencing without simultaneous RNA sequencing may lead to false-negative results for certain viruses. Using DNA- and RNA-based mNGS in BALF, we found 20 viral analytes, 100% of which were coinfections with bacteria and fungi. By directly sequencing clinical specimens, mNGS-based methods may provide more comprehensive information regarding the spectrum of the infections with a single test.

The lungs are not considered sterile, and potentially pathogenic organisms are commonly present in the lungs of healthy asymptomatic individuals (32). The widespread microbes in the environment or DNA contaminants in laboratory reagents, experimental apparatus, and instruments (such as centrifuge tubes) may produce false-positive results during sequence-based pathogen detection (24, 25). Hence, distinguishing causative microbes from background microbiota is a crucial challenge in interpreting mNGS data. Although the read values of mNGS are commonly used for the interpretation of distinct pathogenic infections (33–35), the cutoff reads for diagnosing distinct microbes using mNGS and their clinical significance remain to be clarified. However, in cases of polymicrobial infections, while predominant pathogens are detected, less abundant pathogens may be filtered out by high detection thresholds. Furthermore, misinterpretations caused by the short length of sequencing reads, incomplete databases, improper algorithms, or highly homologous genomes produce false-positive or inconsistent results (36, 37). In contrast, false-negative mNGS results may be attributed to low mapped reads due to the absence of an enrichment procedure for bacterial DNA by the depletion of host nucleic acids (38). More importantly, pathogens that may be considered contaminants, such as *Bacillus* and coagulase-negative Staphylococcus, can lead to severe infections and should not be ignored when interpreting mNGS results (39). When comparing this very sensitive mNGS method with a less sensitive culture method, establishing which results are truly positive, true negative, false-positive, and false-negative is highly challenging. Using an optimized bioinformatics pipeline (13) to identify clinically relevant pathogens based on coverage, depth, reads, and literature evidence of pathogenicity may facilitate the identification of the causative pathogens.

The turnaround time of the mNGS analysis in this study was 48 to 72 h, which could be shorter than culture (2 to 4 days for bacteria or fungi, 7 to 28 days for viruses, and 7 to 56 days for mycobacteria). Using the localization of the hospital site sequencing platform, the turnaround time could be further reduced to <24 h in a recent study (27). Sepsis is a challenge in intensive care medicine, with a high mortality rate and delays in effective antimicrobial therapy resulting in decreased survival. In this study, we showed that mNGS had higher positive rates than culture, especially for bacteria, viruses, and multipathogen infections, which are difficult to culture and detect in patients treated with empirical antibiotics during sepsis. mNGS also showed a high NPA with cultures. Although we found a relatively high failure rate (12%) for shotgun sequencing of RNA, we suggest that the stable turnaround time of mNGS with DNA shotgun sequencing makes this method suitable for clinical practice.

This study has several limitations. First, the blood samples for mNGS and culture were not concomitantly sampled, which may have led to a higher discrepancy between mNGS and culture findings. Furthermore, this study was conducted at a tertiary referral center. Therefore, samples containing rare pathogens may not reflect the actual performance of mNGS in clinically relevant situations. We could not evaluate the association between the number of reads and clinical prognosis because of the relatively small sample size and distinct pathogenic infections. Therefore, further investigation is needed to clarify this issue. Antibiotic resistance genes could not be detected by mNGS. Finally, a previous study (25) found that regardless of the sample being taken from an uninfected or infected individual, >95% of the reads obtained were mapped to the human genome, and <1% were mapped to pathogen genomes. Techniques that exclude host DNA data and improve the percentage of reads for targeted pathogen genomes should be developed to exploit the potential of mNGS.

In conclusion, we showed that mNGS had higher positive rates than culture methods, especially for bacteria and viruses, which are difficult to culture and detect in antibiotic-treated patients. Furthermore, mNGS is a promising method for the detection of multipathogen infections. Nonetheless, the interpretation of mNGS data should be combined with clinical data and conventional diagnostic methods, considering the lack of unified diagnostic criteria.

## MATERIALS AND METHODS

### Study patients and clinical specimens.

From July 2020 to March 2021, we conducted a prospective study at the National Taiwan University Hospital, a tertiary medical center with 2400 beds located in northern Taiwan. A considerable proportion of the enrolled patients were immunocompromised due to cancer, cirrhosis, dialysis, or immunodeficiency. Patients ≥20 years of age with a clinical diagnosis of sepsis were enrolled in this study. Sepsis is defined as an acute infection with a sepsis-related organ failure assessment (SOFA) score ≥2. Acute Physiology and Chronic Health Evaluation (APACHE II) and SOFA scores were calculated upon admission to the ICU. Blood cultures, including two bacterial cultures and one fungal culture, as well as BALF bacterial culture, fungal culture, acid-fast staining with mycobacterial culture, and viral isolation were performed for every enrolled patient. Blood and BALF samples for mNGS were collected within 24 h of the onset of sepsis. This study was approved by the Institutional Review Board and Ethical Committee of the National Taiwan University Hospital (202001051RINC).

### Conventional microbiological cultures and identification.

Blood cultures were performed using Bactec Plus Aerobic/F and Bactec Anaerobic Lytic/10 vials (Becton, Dickinson Microbiology Systems, Sparks, MD, USA) with inoculation of 8 to 10 mL of blood sample in each vial. Positive blood culture vials were subcultured on Trypticase soy agar with 5% sheep blood agar (BAP), chocolate agar, and CDC Anaerobe 5% sheep blood agar with phenylethyl alcohol (Becton, Dickinson Microbiology Systems). For BALF, bacterial cultures were processed by inoculating the sample onto a BAP/eosin methylene blue agar biplate (Becton, Dickinson Microbiology Systems) and chocolate agar, and incubated in an atmosphere enriched with 5% CO2 at 35°C. The culture plates were read after 18 to 24 h and kept for four more days before reporting as negative. Fungal cultures were performed on Sabouraud dextrose agar, Mycocel slant, and inhibitory mold agar slant (Becton, Dickinson Microbiology Systems) for 28 days. For mycobacterial cultures, samples were cultured for 56 days after inoculation into the following two types of media: the Bactec mycobacteria growth indicator tube and the Lowenstein-Jensen medium slant (Becton, Dickinson Microbiology System). The organisms were identified using a matrix-assisted laser desorption/ionization time-of-flight mass spectrometry system (Bruker Biotyper; Bruker Daltonik GmbH, Bremen, Germany). For virus isolation, blood samples were cultured with human embryonic lung cells, and BALF samples were cultured with HeLa, human epithelial cell-2, rhabdomyosarcoma, LLC-rhesus monkey kidney cell-2, and Mardin-Darby canine kidney cells for a maximum of 28 days.

### DNA extraction and library construction.

Nucleic acid (DNA and RNA) extraction was performed in batches with 300 μL of blood and 600 μL of pretreated BALF samples. The library was denatured at 95°C for 6 min to obtain single-stranded DNA and circularized using ligase at 37°C for 30 min. The single-strand circularized library was then transformed into nanoballs at 30°C for 25 min using the DNB polymerase I provided with the DNBSEQ-G400RS FCL SE50 sequencing kit (MGI, Shenzhen, China). The library was then sequenced using the MGISEQ-2000 platform with the DNBSEQ-G400RS sequencing flow cell, resulting in 100 million reads per blood sample and 40 million reads per BALF sample.

### Sequencing and bioinformatic analysis.

High-quality sequencing data were generated by removing short-(<35 bp), low-quality, and low-complexity reads. The reads were then mapped to the human reference genome hg38 (GRCh38, December 2017) using the Burrows-Wheeler Aligner (40). The remaining data were aligned with the Microbial Genome Database using the Burrows-Wheeler Alignment tool (v0.7.10-r789). The classification reference database is composed of 4,945 whole-genome sequences of viral taxa, 6,039 bacterial genomes or scaffolds, 1,064 fungi, 234 parasites, 174 species of Mycobacterium, and 137 Mycoplasma/Chlamydia associated with human infections downloaded from NCBI (https://ncbi.nlm.nih.gov/genome) (41–44). Duplicate reads were removed using PRINSEQ (v.0.20.4) (45). Microorganisms with at least three specific reads and high coverage were considered possible pathogens. The success rates of shotgun DNA and RNA sequencing were 98% and 88%, respectively (Table S4).

### Statistical analyses.

Categorical variables were compared using the Chi-square test or Fisher’s exact test, where appropriate, and differences in continuous variables were analyzed using the independent *t* test or the Mann-Whitney rank-sum test depending on the data distribution. Data are presented as numbers (percentages) or medians (ranges). The PPA was calculated as the proportion of mNGS/culture-positive results in culture results that were positive, and the NPA was calculated as the proportion of mNGS/culture-negative results in culture results that were negative.
