# Supplementary material for: Utility of Metagenomic Next-Generation Sequencing for Etiological Diagnosis of Patients with Sepsis in Intensive Care Units
Source: Microbiol Spectr. 2022 Jul 21;10(4):e00746-22. doi: 10.1128/spectrum.00746-22 (PMC9430677; doi:10.1128/spectrum.00746-22)
Supplement: Supplemental file 1 — Supplemental material. Download spectrum.00746-22-s0001.pdf, PDF file, 1.1 MB [file spectrum.00746-22-s0001.pdf]

Supplementary TABLE 1 Species detected by routine culture and metagenomic next-generation sequencing (mNGS) of blood and bronchial alveolar lavage fluid in each patient

Case No.	Blood				Bronchial alveolar lavage fluid						
	Culture	mNGS (reads)			Culture	mNGS (reads)					
		Bacteria	Fungus	Virus		Bacteria		Fungus		Virus	
		DNA	DNA	DNA		DNA	RNA	DNA	RNA	DNA	RNA
1	Escherichia coli				No growth	Escherichia coli (4)	Prevotella salivae (9)				
						Prevotella salivae (4)	Corynebacterium accolens (7)				
						Prevotella oris (3)	Staphylococcus epidermidis (7)				
							Prevotella pallens (6)				
							Moraxella osloensis (6)				
							Corynebacterium matruchotii (4)				
							Escherichia coli (3)				
2	No growth	Enterococcus hirae (33)			Staphylococcus aureus	Lactobacillus fermentum (2811)	Lactobacillus fermentum (8695)	Candida albicans (105)	Candida albicans (84)		
		Veillonella parvula (15)				Veillonella parvula (848)	Enterococcus hirae (1826)				
		Lactobacillus fermentum (17)				Prevotella salivae (801)	Veillonella parvula (1667)				
		Prevotella melaninogenica (5)				Enterococcus hirae (560)	Prevotella salivae (1488)				
						Atopobium parvulum (500)	Cutibacterium acnes (675)				
						Streptococcus salivarius (493)	Streptococcus salivarius (669)				
						Selenomonas flueggei (133)	Veillonella atypica (593)				
						Megasphaera micronuciformis (115)	Selenomonas flueggei (379)				
						Schaalia odontolytica (88)	Megasphaera micronuciformis (375)				
						Sanguibacter keddieii (40)	Atopobium parvulum (349)				
						Klebsiella pneumoniae (29)	Schaalia odontolytica (266)				
						Actinomyces graevenitzii (12)	Prevotella melaninogenica (200)				
							Sanguibacter keddieii (153)				
							Klebsiella pneumoniae (64)				
							Propionibacterium humerusii (26)				
							Selenomonas noxia (12)				
							Streptococcus mitis (10)				
3	No growth				Stenotrophomonas maltophilia	Stenotrophomonas maltophilia (18035)	Stenotrophomonas maltophilia (30650)	Candida parapsilosis (232)	Candida parapsilosis (88)	CMV (55)	HSV1 (19)
						Cutibacterium acnes (6)	Cutibacterium acnes (355)	Candida albicans (98)	Candida albicans (77)	HSV1 (7)	CMV (6)
						Klebsiella pneumoniae (2)	Staphylococcus epidermidis (31)				
							Staphylococcus warneri (27)				
							Tannerella forsythia (27)				
							Corynebacterium accolens (26)				
							Veillonella parvula (18)				
							Propionibacterium humerusii (15)				
							Streptococcus oralis (10)				
							Bifidobacterium thermophilum (7)				
							Haemophilus parainfluenzae (6)				
							Staphylococcus aureus (4)				
4	No growth				No growth	Cutibacterium acnes (6)	Cutibacterium acnes (2132)	Schizophyllum commune (8715)	Schizophyllum commune (578)		
							Corynebacterium accolens (182)				
							Staphylococcus epidermidis (89)				
							Propionibacterium humerusii (50)				
							Bacteroides uniformis (35)				
							Corynebacterium aurimucosum (31)				
							Staphylococcus aureus (17)				
							Streptococcus mitis (15)				
							Moraxella osloensis (15)				
							Prevotella copri (12)				
							Staphylococcus warneri (11)				
							Citrobacter koseri (8)				
							Serratia marcescens (5)				
5	No growth				Mixed flora	Corynebacterium jeikeium (754)	Corynebacterium jeikeium (3936)			EBV (8)	EBV (22)
						Corynebacterium resistens (176)	Corynebacterium resistens (956)				
						Staphylococcus haemolyticus (89)	Staphylococcus haemolyticus (303)				
						Staphylococcus epidermidis (12)	Cutibacterium acnes (169)				
						Veillonella parvula (10)	Veillonella parvula (61)				
						Cutibacterium acnes (8)	Staphylococcus epidermidis (28)				
						Fusobacterium nucleatum (4)	Fusobacterium nucleatum (17)				
							Mycoplasma orale (15)				
							Propionibacterium humerusii (7)				
6	No growth	Staphylococcus epidermidis (149)	Aspergillus versicolor (42)	EBV (6)	Mixed flora	Klebsiella variicola (2405)	Klebsiella variicola (4413)				
		Acinetobacter johnsonii (48)	Aureobasidium pullulans (13)			Acinetobacter nosocomialis (215)	Acinetobacter nosocomialis (279)				
		Staphylococcus warneri (35)				Klebsiella pneumoniae (92)	Klebsiella pneumoniae (212)				
						Corynebacterium striatum (64)	Cutibacterium acnes (176)				
						Burkholderia cenocepacia (35)	Corynebacterium striatum (92)				
						Enterococcus faecium (11)	Burkholderia cenocepacia (45)				
							Enterococcus faecium (21)				
							Enterobacter cloacae (1)				
							Escherichia coli (1)				
7	Klebsiella pneumoniae	Prevotella oris (33)		HPV16 (572)	Pseudomonas aeruginosa	Pseudomonas aeruginosa (2943)	Pseudomonas aeruginosa (4799)			EBV (38)	EBV (88)
	Morganella morganii	Cutibacterium acnes (25)		CMV (271)	Acinetobacter baumannii	Streptococcus anginosus (243)	Streptococcus anginosus (1140)				CMV (8)
	Streptococcus anginosus	Streptococcus anginosus (23)		EBV (20)		Prevotella oris (181)	Prevotella oris (789)				
		Enterobacter hormaechei (18)		JC polyomavirus (13)		Elizabethkingia anophelis (113)	Haemophilus influenzae (310)				
		Morganella morganii (16)		BK polyomavirus1 (7)		Staphylococcus aureus (87)	Acinetobacter pittii (295)				
		Pseudomonas aeruginosa (12)				Mycoplasma orale (64)	Staphylococcus aureus (168)				
		Enterobacter cloacae (4)				Prevotella melaninogenica (52)	Fusobacterium nucleatum (111)				
		Klebsiella pneumoniae (4)				Neisseria mucosa (48)	Elizabethkingia anophelis (105)				
		Escherichia coli (2)				Haemophilus influenzae (45)	Klebsiella pneumoniae (16)				
						Fusobacterium nucleatum (24)	Escherichia coli (9)				
						Alloprevotella tannerae (18)	Citrobacter koseri (8)				
						Morganella morganii (13)					
						Neisseria sicca (10)					
						Klebsiella pneumoniae (7)					
						Bifidobacterium dentium (6)					
						Lactobacillus crispatus (6)					
						Alloprevotella rava (5)					
8	No growth			CMV (67)	No growth	Veillonella dispar (382)	Veillonella dispar (324)				CMV (5)
						Fusobacterium periodonticum (160)	Chryseobacterium indologenes (161)				
						Prevotella pallens (105)	Prevotella pallens (106)				
						Chryseobacterium indologenes (59)	Streptococcus oralis (58)				
						Streptococcus oralis (56)	Arcobacter cryaerophilus (15)				

9	No growth	Staphylococcus hominis (494)			Mixed flora	Acinetobacter baumannii (8128)	Acinetobacter baumannii (7112)				
		Haemophilus parainfluenzae (27)				Corynebacterium striatum (2140)	Corynebacterium striatum (3565)				
						Fusobacterium nucleatum (353)	Cutibacterium acnes (1289)				
						Porphyromonas gingivalis (329)	Porphyromonas gingivalis (374)				
						Streptococcus anginosus (47)	Fusobacterium nucleatum (259)				
						Haemophilus parainfluenzae (24)	Haemophilus parainfluenzae (25)				
						Enterococcus faecalis (11)	Enterococcus faecalis (14)				
						Klebsiella pneumoniae (4)	Staphylococcus aureus (10)				
						Bacteroides uniformis (4)	Citrobacter koseri (8)				
							Klebsiella pneumoniae (3)				
10	No growth	Acinetobacter johnsonii (55)	Aspergillus turcosus (7)		Candida albicans	Stenotrophomonas maltophilia (4)	Cutibacterium acnes (137)				
		Staphylococcus epidermidis (15)				Cutibacterium acnes (3)	Moraxella osloensis (3)				
		Staphylococcus hominis (10)					Pseudomonas aeruginosa (2)				
11	No growth	Helicobacter pylori (10)	Cladosporium sphaerospermum (103)		Achromobacter xylosoxidans	Achromobacter xylosoxidans (835)	Achromobacter xylosoxidans (2656)				
		Staphylococcus epidermidis (5)			Mycobacterium avium	Cutibacterium acnes (3)	Cutibacterium acnes (21)				
		Staphylococcus hominis (5)									
12	No growth	Klebsiella quasipneumoniae (2079)	Candida albicans (4)	EBV (46)	Acinetobacter nosocomialis	Klebsiella quasipneumoniae (20750)	Klebsiella quasipneumoniae (96196)	Candida tropicalis (12)	Candida tropicalis (19)		HSV1 (41)
		Enterococcus faecium (72)			Klebsiella pneumoniae	Enterococcus faecium (3247)	Enterococcus faecium (24053)				
		Klebsiella pneumoniae (31)				Staphylococcus epidermidis (1159)	Klebsiella pneumoniae (3681)				
		Staphylococcus warneri (8)				Klebsiella pneumoniae (681)	Veillonella parvula (1329)				
		Staphylococcus hominis (6)				Acinetobacter nosocomialis (69)	Haemophilus parainfluenzae (856)				
						Corynebacterium striatum (67)	Bacteroides uniformis (793)				
						Veillonella parvula (51)	Streptococcus oralis (734)				
						Lactobacillus paracasei (15)	Prevotella intermedia (487)				
						Pseudomonas aeruginosa (7)	Fusobacterium nucleatum (448)				
							Streptococcus sanguinis (359)				
							Anaerococcus prevotii (281)				
							Staphylococcus aureus (159)				
							Pseudomonas aeruginosa (62)				
							Streptococcus pneumoniae (41)				
							Escherichia coli (15)				
							Elizabethkingia meningoseptica (9)				
							Enterobacter cloacae (6)				
							Citrobacter koseri (3)				
13	No growth	Methylorubrum extorquens (8)		VZV (35)	Acinetobacter baumannii	Acinetobacter baumannii (206)	Acinetobacter baumannii (8882)		Candida parapsilosis (7)		
		Enterococcus faecium (7)				Cutibacterium acnes (90)	Bacteroides uniformis (155)				
						Moraxella osloensis (28)	Pseudomonas aeruginosa (32)				
						Staphylococcus epidermidis (12)	Enterococcus faecium (27)				
						Corynebacterium callunae (12)	Haemophilus parainfluenzae (20)				
							Staphylococcus aureus (16)				
							Escherichia coli (12)				
							Stenotrophomonas maltophilia (11)				
							Serratia marcescens (8)				
							Citrobacter koseri (8)				
							Klebsiella pneumoniae (2)				
							Streptococcus pneumoniae (1)				
14	Enterobacter cloacae complex	Enterobacter hormaechei (27190)		CMV (2298)	Enterobacter cloacae complex	Enterobacter hormaechei (33322)	Enterobacter hormaechei (35136)			CMV (19)	CMV (41)
		Klebsiella quasipneumoniae (7191)		EBV (6)	Klebsiella pneumoniae	Klebsiella quasipneumoniae (7520)	Klebsiella quasipneumoniae (8353)			EBV (12)	EBV (28)
		Enterobacter cloacae (280)			CMV	Enterococcus faecalis (533)	Enterococcus faecalis (1271)				
		Enterococcus faecalis (102)				Enterobacter cloacae (375)	Weissella confusa (919)				
		Klebsiella pneumoniae (72)				Weissella confusa (363)	Corynebacterium resistens (655)				
		Stenotrophomonas maltophilia (36)				Corynebacterium resistens (352)	Enterobacter cloacae (406)				
		Citrobacter freundii (31)				Stenotrophomonas maltophilia (100)	Stenotrophomonas maltophilia (111)				
		Methylorubrum extorquens (21)				Klebsiella pneumoniae (70)	Klebsiella pneumoniae (106)				
		Staphylococcus epidermidis (11)				Citrobacter freundii (35)	Citrobacter freundii (43)				
		Cutibacterium acnes (11)					Pseudomonas aeruginosa (2)				
		Sphingomonas echinoides (5)									
15	Staphylococcus aureus	Enterobacter hormaechei (13329)	Aspergillus turcosus (6)		Candida albicans	Enterobacter hormaechei (52296)					
		Staphylococcus aureus (395)	Cladosporium sphaerospermum (5)		Mixed flora	Veillonella parvula (5618)					
		Citrobacter youngae (317)				Klebsiella aerogenes (703)					
		Klebsiella pneumoniae (299)				Citrobacter youngae (688)					
		Klebsiella aerogenes (297)				Haemophilus parainfluenzae (493)					
		Enterobacter cloacae (294)				Klebsiella pneumoniae (391)					
		Prevotella melaninogenica (260)				Prevotella melaninogenica (143)					
		Haemophilus parainfluenzae (192)				Staphylococcus aureus (118)					
		Veillonella parvula (179)				Streptococcus anginosus (111)					
		Streptococcus anginosus (139)				Lactobacillus salivarius (88)					
		Streptococcus constellatus (59)				Fusobacterium nucleatum (80)					
		Fusobacterium nucleatum (49)				Citrobacter freundii (41)					
		Prevotella oris (47)				Escherichia coli (3)					
		Cutibacterium acnes (29)									
		Citrobacter freundii (20)									
		Methylorubrum extorquens (19)									
		Moraxella osloensis (13)									
		Staphylococcus hominis (6)									
		Escherichia coli (1)									
17	No growth	Acinetobacter johnsonii (30)			Mycobacterium mantenii	Cutibacterium acnes (40)	Cutibacterium acnes (102)	Malassezia restricta (7)			
						Moraxella osloensis (7)	Streptococcus pseudopneumoniae (8)	Aspergillus turcosus (6)			
						Propionibacterium humerusii (3)	Veillonella parvula (7)				
18	Candida glabrata	Ralstonia pickettii (46)	Aspergillus turcosus (10)	EBV (47)	Candida albicans	Burkholderia cenocepacia (469)	Enterococcus raffinosus (361)	Candida albicans (8)	Candida albicans (50)		
		Methylorubrum extorquens (41)			Mixed flora	Enterococcus raffinosus (22)	Burkholderia cenocepacia (170)	Candida glabrata (7)			

19	No growth				Trichosporon asahii	Ralstonia mannitolilytica (246)	Enterococcus faecium (80)	Trichosporon asahii (263)		HSV1 (25866)	HSV1 (39457)
					Mixed flora	Staphylococcus haemolyticus (33)	Staphylococcus haemolyticus (51)			EBV (17)	EBV (13)
						Enterococcus faecium (14)	Acinetobacter nosocomialis (7)				
						Acinetobacter nosocomialis (4)					
20	No growth	Ralstonia pickettii (856)			Candida albicans			Candida albicans (17)	Candida albicans (19)		
		Bradyrhizobium elkanii (296)			Mixed flora						
21	No growth	Ralstonia pickettii (76)		Human betaherpesvirus 6B (17)	Candida albicans						
		Bradyrhizobium elkanii (10)									
22	No growth	Methyloversatilis discipulorum (526)		EBV (10)	Candida albicans			Candida albicans (24)		EBV (7)	
		Acinetobacter johnsonii (477)									
23	No growth			HSV1 (107)	Candida albicans	Streptococcus anginosus (184)	Streptococcus anginosus (7932)	Candida albicans (10)	Candida albicans (295)	HSV1 (31872)	HSV1 (42775)
					HSV1	Lactobacillus gasseri (33)	Lactobacillus gasseri (1072)				
					Mixed flora						
24	No growth	Acinetobacter johnsonii (164)	Candida parapsilosis (250)		Mycobacterium tuberculosis	Porphyromonas endodontalis (12)					
					Mixed flora						
25	No growth	Neisseria flavescens (97)	Melampsora pinitorqua (24)	Human betaherpesvirus 7 (4)	Candida glabrata	Streptococcus mutans (306)	Lactobacillus fermentum (53)				
		Haemophilus parainfluenzae (78)			Mycobacterium avium	Lactobacillus fermentum (8)	Streptococcus mutans (38)				
					Mixed flora						
26	No growth	Serratia marcescens (2800)			Serratia ureilytica	Serratia marcescens (1160)					
		Alloprevotella tannerae (446)			Pseudomonas aeruginosa	Enterococcus faecalis (673)					
		Enterococcus faecalis (198)			Candida albicans	Alloprevotella tannerae (270)					
		Acinetobacter nosocomialis (114)				Acinetobacter nosocomialis (102)					
		Haemophilus parainfluenzae (86)				Haemophilus parainfluenzae (83)					
		Klebsiella aerogenes (81)				Streptococcus agalactiae (39)					
		Streptococcus agalactiae (9)				Klebsiella aerogenes (26)					
						Pseudomonas aeruginosa (16)					
27	Cryptococcus neoformans	Prevotella melaninogenica (24)	Pneumocystis jirovecii (934)	CMV (1949)	Acinetobacter nosocomialis	Acinetobacter nosocomialis (7605)	Staphylococcus haemolyticus (51867)	Pneumocystis jirovecii (75)		HSV1 (5103)	HSV1 (22856)
	Corynebacterium species			Human polyomavirus 1 (14)	Stenotrophomonas maltophilia	Staphylococcus haemolyticus (5874)	Acinetobacter nosocomialis (15657)			CMV (79)	CMV (1217)
				HSV1 (10)	Candida haemulonii	Stenotrophomonas maltophilia (1140)	Serratia marcescens (982)				
						Serratia marcescens (335)	Stenotrophomonas maltophilia (760)				
						Corynebacterium pseudogenitalium (226)					
28	No growth	Acinetobacter johnsonii (231)			No growth	Tropheryma whipplei (25)	Tropheryma whipplei (24)				
		Moraxella osloensis (98)					Corynebacterium matruchotii (13)				
29	No growth	Ralstonia pickettii (363)	Candida albicans (25)	EBV (23)	Candida albicans	Ralstonia mannitolilytica (626)	Ralstonia mannitolilytica (25945)	Candida albicans (45058)	Candida albicans (2309)	HSV1 (139)	HSV1 (61)
				HSV1 (14)	Mixed flora	Haemophilus parahaemolyticus (43)	Acinetobacter nosocomialis (6068)				
						Neisseria flavescens (32)	Haemophilus parainfluenzae (28)				
						Acinetobacter nosocomialis (16)					
30	No growth	Klebsiella pneumoniae (3790)	Candida glabrata (11)	VZV (6934)	Pseudomonas aeruginosa	Klebsiella pneumoniae (1428)		Candida albicans (5)		CMV (19)	
		Lactobacillus mucosae (1093)		JC polyomavirus (37)	Klebsiella pneumoniae	Lactobacillus mucosae (314)					
		Pseudomonas aeruginosa (320)		CMV (22)	Candida glabrata	Acinetobacter baumannii (286)					
		Enterobacter cloacae (211)		EBV (11)		Pseudomonas aeruginosa (210)					
		Enterococcus faecium (58)				Enterobacter cloacae (102)					
		Citrobacter freundii (57)				Citrobacter freundii (72)					
		Escherichia coli (38)				Corynebacterium striatum (68)					
		Chryseobacterium indologenes (33)				Enterococcus faecalis (58)					
						Escherichia coli (13)					
						Stenotrophomonas maltophilia (12)					
31	No growth	Ralstonia pickettii (101)			Mixed flora	Veillonella dispar (116)	Veillonella atypica (403)				
		Acinetobacter johnsonii (72)				Fusobacterium periodonticum (86)	Streptococcus parasanguinis (277)				
						Haemophilus parainfluenzae (58)	Haemophilus parainfluenzae (191)				
						Streptococcus parasanguinis (53)	Neisseria flavescens (50)				
32	Pseudomonas aeruginosa	Ralstonia pickettii (93)			Pseudomonas aeruginosa	Pseudomonas aeruginosa (10707)	Pseudomonas aeruginosa (18075)				
		Pseudomonas aeruginosa (83)				Eggerthia catenaformis (33)	Eggerthia catenaformis (58)				
						Achromobacter insuavis (6)	Achromobacter insuavis (39)				
33	No growth		Candida parapsilosis (17)		Serratia marcescens	Tannerella forsythia (4331)					
						Porphyromonas gingivalis (1674)					
						Filifactor alocis (1043)					
						Campylobacter rectus (945)					
						Corynebacterium striatum (21)					
34	No growth	Klebsiella pneumoniae (128)		CMV (2430)	Klebsiella pneumoniae	Klebsiella pneumoniae (28669)				CMV (5)	
		Stenotrophomonas maltophilia (74)		EBV (78)		Stenotrophomonas maltophilia (148)					
		Bacteroides uniformis (18)		JC polyomavirus (68)		Staphylococcus haemolyticus (94)					
				Human alphaherpesvirus 2 (31)							
				Human polyomavirus 1 (26)		Streptococcus mitis (19)					
36	No growth	Enterococcus faecium (107)		CMV (72)	Klebsiella aerogenes	Enterococcus faecium (4765)	Enterococcus faecium (40814)			HSV1 (9)	HSV1 (84)
		Klebsiella aerogenes (61)		EBV (11)	Pseudomonas aeruginosa	Klebsiella aerogenes (2102)	Klebsiella aerogenes (26728)				CMV (7)
		Enterobacter hormaechei (19)				Enterobacter hormaechei (332)	Enterobacter hormaechei (4440)				
						Pseudomonas aeruginosa (12)	Pseudomonas aeruginosa (239)				

37	No growth			HSV1 (93)	Candida albicans		Fusobacterium nucleatum (3452)		Candida albicans (36)		HSV1 (434)
				EBV (9)	Mixed flora		Streptococcus intermedius (2351)				
							Haemophilus influenzae (269)				
38	No growth			Trichodysplasia spinulosa-associated polyomavirus (5084)	No growth	Ralstonia mannitolilytica (101)	Ralstonia mannitolilytica (1072)				Trichodysplasia spinulosa-associated polyomavirus (12)
				Human gammaherpesvirus 8 (54)		Burkholderia ubonensis (3)	Acinetobacter baumannii (82)				
39	No growth	Staphylococcus haemolyticus (271)			No growth						
40	Staphylococcus aureus	Acinetobacter johnsonii (118)		CMV (35)	Staphylococcus aureus	Staphylococcus aureus (1243)	Staphylococcus aureus (1459)				
		Staphylococcus aureus (95)			Escherichia coli	Escherichia coli (230)	Escherichia coli (782)				
		Escherichia coli (15)				Streptococcus parasanguinis (15)	Streptococcus parasanguinis (37)				
						Enterobacter hormaechei (12)	Enterobacter hormaechei (15)				
41	Escherichia coli	Acinetobacter johnsonii (522)			Escherichia coli	Escherichia coli (230)	Escherichia coli (3131)				
		Escherichia coli (184)			Candida albicans	Pseudomonas putida (62)	Burkholderia cenocepacia (76)				
						Delftia acidovorans (12)					
						Burkholderia cenocepacia (3)					
42	Enterobacter cloacae complex	Pseudomonas putida (72)		CMV (76)	Staphylococcus aureus	Prevotella melaninogenica (569)	Prevotella melaninogenica (644)	Pneumocystis jirovecii (6)			CMV (7)
	Klebsiella pneumoniae	Staphylococcus argenteus (38)			Klebsiella pneumoniae	Fusobacterium nucleatum (419)	Fusobacterium nucleatum (409)				
	Staphylococcus aureus	Enterococcus faecalis (10)			Candida tropicalis	Streptococcus anginosus (354)	Streptococcus constellatus (319)				
		Klebsiella pneumoniae (9)			CMV	Streptococcus constellatus (335)	Streptococcus anginosus (216)				
		Enterobacter hormaechei (5)				Staphylococcus argenteus (253)	Staphylococcus argenteus (154)				
						Parvimonas micra (201)	Parvimonas micra (32)				
						Klebsiella pneumoniae (37)	Klebsiella pneumoniae (24)				
						Enterobacter hormaechei (19)	Enterobacter hormaechei (18)				
							Campylobacter rectus (8)				
43	No growth	Streptococcus mitis (250)	Candida tropicalis (17)	CMV (2104)	Candida tropicalis	Enterobacter hormaechei (5056)	Enterobacter hormaechei (6148)			CMV (80)	CMV (148)
		Enterococcus faecium (1165)		EBV (4189)	Mixed flora	Acinetobacter nosocomialis (4487)	Acinetobacter nosocomialis (2850)			Human polyomavirus 1 (9)	
						Stenotrophomonas maltophilia (2948)	Stenotrophomonas maltophilia (2650)				
						Ralstonia mannitolilytica (195)	Ralstonia mannitolilytica (313)				
						Corynebacterium resistens (82)	Corynebacterium resistens (114)				
						Citrobacter freundii (3)					
44	Pseudomonas aeruginosa	Proteus mirabilis (7742)		Human betaherpesvirus 6B (187)	Candida tropicalis	Proteus mirabilis (8808)	Proteus mirabilis (23871)				
		Pseudomonas aeruginosa (3051)		CMV (24)	Mixed flora	Pseudomonas aeruginosa (1449)	Pseudomonas aeruginosa (10481)				
		Klebsiella pneumoniae (955)				Klebsiella pneumoniae (993)	Klebsiella pneumoniae (4365)				
						Prevotella oris (23)	Prevotella oris (25)				
						Enterococcus avium (7)					
45	No growth	Acinetobacter johnsonii (758)		CMV (42)	Candida albicans	Pseudomonas putida (753)		Candida albicans (1164)	Candida albicans (303)	HSV1 (927)	HSV1 (2942)
		Pseudomonas putida (161)			Mixed flora	Delftia tsuruhatensis (161)					Human betaherpesvirus 7 (3)
		Staphylococcus capitis (63)									
		Streptococcus mitis (22)									
46	No growth				Mixed flora	Corynebacterium propinquum (347)					Human RSVA (1)
						Staphylococcus haemolyticus (11)					
						Neisseria flavescens (3)					
47	No growth	Acinetobacter johnsonii (571)			Candida albicans	Fusobacterium nucleatum (19725)	Fusobacterium nucleatum (3939)	Candida albicans (584)	Candida albicans (61)		
		Streptococcus mitis (49)			Mixed flora	Cutibacterium acnes (38)	Cutibacterium acnes (67)				
						Pseudomonas aeruginosa (8)					
48	No growth	Acinetobacter johnsonii (37)		Human betaherpesvirus 6B (5)	Klebsiella pneumoniae	Klebsiella pneumoniae (291)	Klebsiella pneumoniae (296)	Candida albicans (116)			
		Ralstonia pickettii (35)			Candida albicans	Streptococcus constellatus (73)	Streptococcus constellatus (76)				
		Pseudomonas putida (33)				Acinetobacter nosocomialis (71)	Serratia marcescens (48)				
		Delftia tsuruhatensis (15)				Serratia marcescens (43)	Acinetobacter nosocomialis (45)				
		Klebsiella pneumoniae (10)				Ralstonia mannitolilytica (19)	Pseudomonas aeruginosa (27)				
						Pseudomonas aeruginosa (15)					
49	Staphylococcus capitis	Acinetobacter johnsonii (378)		Hepatitis B virus (2)	Acinetobacter baumannii	Acinetobacter baumannii (9871)	Acinetobacter baumannii (30093)	Candida albicans (5)			
	Staphylococcus epidermidis	Mycobacterium lentiflavum (1)				Pseudomonas putida (236)					
	Candida parapsilosis complex					Mycobacterium lentiflavum (2)					
50	Klebsiella pneumoniae	Pseudomonas aeruginosa (235)			Pseudomonas aeruginosa	Pseudomonas aeruginosa (313)	Pseudomonas aeruginosa (1041)				
	Pseudomonas aeruginosa	Klebsiella pneumoniae (81)			Proteus vulgaris	Bacteroides fragilis (210)	Bacteroides fragilis (434)				
		Bacteroides fragilis (63)				Klebsiella pneumoniae (41)	Klebsiella pneumoniae (284)				
		Proteus vulgaris (6)									
51	No growth	Acinetobacter johnsonii (512)			Mixed flora						
		Pseudomonas putida (166)									
52	No growth	Staphylococcus hominis (99)		Human gammaherpesvirus 8 (29)	No growth						

Supplementary TABLE 2 Species detected by routine culture and metagenomic next-generation sequencing (mNGS) of blood and bronchial alveolar lavage fluid

	Blood		Bronchial alveolar lavage fluid			
Species[#]	Culture	mNGS (DNA)	Culture	mNGS (DNA)	mNGS (RNA)	mNGS (DNA/RNA)
**Total**	21	200	58	254	247	348
**Bacteria, Gram-negative**	11	99	27	139	127	187
Achromobacter insuavis	0	0	0	1	1	1
Acinetobacter johnsonii	0	14	0	0	0	0
Achromobacter xylosoxidans	0	0	1	1	1	1
Acinetobacter baumannii	0	0	3	4	4	5
Acinetobacter nosocomialis	0	1	2	8	6	8
Acinetobacter pittii	0	0	0	0	1	1
Alloprevotella rava	0	0	0	1	0	1
Alloprevotella tannerae	0	1	0	2	0	2
Arcobacter cryaerophilus	0	0	0	0	1	1
Bacteroides fragilis	0	1	0	1	1	1
Bacteroides uniformis	0	1	0	1	3	4
Bifidobacterium dentium	0	0	0	1	0	1
Bradyrhizobium elkanii	0	2	0	0	0	0
Burkholderia cenocepacia	0	0	0	3	3	3
Burkholderia ubonensis	0	0	0	1	0	1
Campylobacter rectus	0	0	0	1	1	2
Chryseobacterium indologenes	0	1	0	1	1	1
Citrobacter freundii	0	3	0	4	1	4
Citrobacter koseri	0	0	0	0	5	5
Citrobacter youngae	0	1	0	1	0	1
Delftia acidovorans	0	0	0	1	0	1
Delftia tsuruhatensis	0	1	0	1	0	1
Elizabethkingia anophelis	0	0	0	1	1	1
Elizabethkingia meningoseptica	0	0	0	0	1	1
Enterobacter cloacae	2	4	1	2	3	4
Enterobacter hormaechei	0	5	0	6	5	6
Escherichia coli	2	5	2	5	7	9
Fusobacterium nucleatum	0	1	0	6	7	8
Fusobacterium periodonticum	0	0	0	2	0	2
Haemophilus influenzae	0	0	0	1	2	2
Haemophilus parahaemolyticus	0	0	0	1	0	1
Haemophilus parainfluenzae	0	4	0	4	6	8
Helicobacter pylori	0	1	0	0	0	0
Klebsiella aerogenes	0	3	1	3	1	3
Klebsiella pneumoniae	3	10	6	14	11	15
Klebsiella quasipneumoniae	0	2	0	2	2	2
Klebsiella variicola	0	0	0	1	1	1
Megasphaera micronuciformis	0	0	0	1	1	1

Methylorubrum extorquens	0	4	0	0	0	0
Methyloversatilis discipulorum	0	1	0	0	0	0
Moraxella osloensis	0	2	0	2	3	5
Morganella morganii	1	1	0	1	0	1
Mycoplasma orale	0	0	0	1	1	2
Neisseria flavescens	0	1	0	2	1	3
Neisseria mucosa	0	0	0	1	0	1
Neisseria sicca	0	0	0	1	0	1
Porphyromonas endodontalis	0	0	0	1	0	1
Porphyromonas gingivalis	0	0	0	2	1	2
Prevotella copri	0	0	0	0	1	1
Prevotella intermedia	0	0	0	0	1	1
Prevotella melaninogenica	0	3	0	3	2	4
Prevotella oris	0	2	0	3	2	3
Prevotella pallens	0	0	0	1	2	2
Prevotella salivae	0	0	0	2	2	2
Proteus mirabilis	0	1	0	1	1	1
Proteus vulgaris	0	1	1	0	0	0
Pseudomonas aeruginosa	3	5	6	10	10	13
Pseudomonas putida	0	4	0	3	0	3
Ralstonia mannitolilytica	0	0	0	5	3	5
Ralstonia pickettii	0	7	0	0	0	0
Selenomonas flueggei	0	0	0	1	1	1
Selenomonas noxia	0	0	0	0	1	1
Serratia marcescens	0	1	1	3	4	5
Sphingomonas echinoides	0	1	0	0	0	0
Serratia ureilytica	0	0	1	0	0	0
Stenotrophomonas maltophilia	0	2	2	7	5	8
Tannerella forsythia	0	0	0	1	1	2
Veillonella atypica	0	0	0	0	2	2
Veillonella dispar	0	0	0	2	1	2
Veillonella parvula	0	2	0	4	5	6
**Bacteria, Gram-positive**	7	39	3	75	83	110
Actinomyces graevenitzii	0	0	0	1	0	1
Anaerococcus prevotii	0	0	0	0	1	1
Atopobium parvulum	0	0	0	1	1	1
Bifidobacterium thermophilum	0	0	0	0	1	1
Corynebacterium species	1	0	0	0	0	0
Corynebacterium accolens	0	0	0	0	3	3
Corynebacterium aurimucosum	0	0	0	0	1	1
Corynebacterium callunae	0	0	0	1	0	1
Corynebacterium jeikeium	0	0	0	1	1	1
Corynebacterium matruchotii	0	0	0	0	2	2
Corynebacterium propinquum	0	0	0	1	0	1
Corynebacterium pseudogenitalium	0	0	0	1	0	1
Corynebacterium resistens	0	0	0	3	3	3
Corynebacterium striatum	0	0	0	5	2	5
Cutibacterium acnes	0	3	0	8	10	11

Eggerthia catenaformis	0	0	0	1	1	1
Enterococcus avium	0	0	0	1	0	1
Enterococcus faecalis	0	3	0	4	2	4
Enterococcus faecium	0	5	0	4	5	5
Enterococcus hirae	0	1	0	1	1	1
Enterococcus raffinosus	0	0	0	1	1	1
Filifactor alocis	0	0	0	1	0	1
Lactobacillus crispatus	0	0	0	1	0	1
Lactobacillus fermentum	0	1	0	2	2	2
Lactobacillus gasseri	0	0	0	1	1	1
Lactobacillus mucosae	0	1	0	1	0	1
Lactobacillus paracasei	0	0	0	1	0	1
Lactobacillus salivarius	0	0	0	1	0	1
Mycobacterium lentiflavum	0	1	0	1	0	1
Parvimonas micra	0	0	0	1	1	1
Propionibacterium humerusii	0	0	0	1	4	5
Sanguibacter keddieii	0	0	0	1	1	1
Schaalia odontolytica	0	0	0	1	1	1
Staphylococcus argenteus	0	1	0	1	1	1
Staphylococcus aureus	3	2	3	3	7	8
Staphylococcus capitis	1	1	0	0	0	0
Staphylococcus epidermidis	1	4	0	3	4	6
Staphylococcus haemolyticus	0	1	0	5	3	5
Staphylococcus hominis	0	6	0	0	0	0
Staphylococcus warneri	0	2	0	0	2	2
Streptococcus agalactiae	0	1	0	1	0	1
Streptococcus anginosus	1	2	0	5	3	5
Streptococcus constellatus	0	1	0	2	2	2
Streptococcus intermedius	0	0	0	0	1	1
Streptococcus mitis	0	3	0	1	2	3
Streptococcus mutans	0	0	0	1	1	1
Streptococcus oralis	0	0	0	1	3	3
Streptococcus parasanguinis	0	0	0	2	2	2
Streptococcus pneumoniae	0	0	0	0	2	2
Streptococcus pseudopneumoniae	0	0	0	0	1	1
Streptococcus salivarius	0	0	0	1	1	1
Streptococcus sanguinis	0	0	0	0	1	1
Tropheryma whipplei	0	0	0	1	1	1
Weissella confusa	0	0	0	1	1	1
**Fungus**	3	15	21	21	13	23
Aureobasidium pullulans	0	1	0	0	0	0
Aspergillus turcosus	0	3	0	1	0	1
Aspergillus versicolor	0	1	0	0	0	0
Candida albicans	0	2	14	12	9	13
Candida glabrata	1	1	2	1	0	1
Candida haemulonii	0	0	1	0	0	0
Candida parapsilosis	1	2	0	1	2	2
Candida tropicalis	0	1	3	1	1	1

Cladosporium sphaerospermum	0	2	0	0	0	0
Cryptococcus neoformans_var_grubii	1	0	0	0	0	0
Malassezia restricta	0	0	0	1	0	1
Melampsora pinitorqua	0	1	0	0	0	0
Pneumocystis jirovecii	0	1	0	2	0	2
Schizophyllum commune	0	0	0	1	1	1
Trichosporon asahii	0	0	1	1	0	1
**Virus**	0	46	3	19	24	28
BK polyomavirus 1	0	1	0	0	0	0
CMV	0	12	2	6	8	10
EBV	0	12	0	5	4	5
HPV16	0	1	0	0	0	0
HSV1	0	4	1	7	9	9
Hepatitis B virus	0	1	0	0	0	0
Human beta-herpesvirus 6B	0	3	0	0	0	0
Human alpha-herpesvirus 2	0	1	0	0	0	0
Human beta-herpesvirus 7	0	1	0	0	1	1
Human gamma-herpesvirus 8	0	2	0	0	0	0
Human polyomavirus 1	0	2	0	1	0	1
Human RSVA	0	0	0	0	1	1
JC polyomavirus	0	3	0	0	0	0
Trichodysplasia spinulosa-associated polyomavirus	0	1	0	0	1	1
VZV	0	2	0	0	0	0
**Mycobacteria**	0	0	4	0	0	0
Mycobacterium avium	0	0	2	0	0	0
Mycobacterium mantenii	0	0	1	0	0	0
Mycobacterium tuberculosis	0	0	1	0	0	0
**Other**						
Toxoplasma gondii	0	1	0	0	0	0

[#]Species marked in bold indicate increased mNGS sensitivity after additional RNA sequencing. BK virus, human polyomavirus; CMV, cytomegalovirus; EBV, Epstein-Barr virus; HPV16, human papillomavirus type 16; HSV, Herpes simplex virus; RSVA, respiratory syncytial virus subtype A VZV, varicella-zoster virus.

Supplementary TABLE 3 Agreement between routine culture and mNGS detection

Species	Culture(+) / mNGS(-)	Culture(+) / mNGS(+)	Culture(-) / mNGS(+)	Culture(-) / mNGS(-)	PPA (%)	NPA (%)
**Bacteria, Gram-negative**	6	32	254	6708	84.2	96.4
Klebsiella pneumoniae	0	9	16	75	100.0	82.4
Pseudomonas aeruginosa	0	9	9	82	100.0	90.1
Escherichia coli	1	3	11	85	75.0	88.5
Acinetobacter johnsonii	0	0	14	86	-	86.0
Haemophilus parainfluenzae	0	0	12	88	-	88.0
Enterobacter hormaechei	0	0	11	89	-	89.0
Stenotrophomonas maltophilia	0	2	8	90	100.0	91.8
Enterobacter cloacae	1	2	6	91	66.7	93.8
Acinetobacter nosocomialis	0	2	7	91	100.0	92.9
Fusobacterium nucleatum	0	0	9	91	-	91.0
Veillonella parvula	0	0	8	92	-	92.0
Serratia marcescens	1	0	6	93	0.0	93.9
Citrobacter freundii	0	0	7	93	-	93.0
Prevotella melaninogenica	0	0	7	93	-	93.0
Pseudomonas putida	0	0	7	93	-	93.0
Moraxella osloensis	0	0	7	93	-	93.0
Ralstonia pickettii	0	0	7	93	-	93.0
Acinetobacter baumannii	1	2	3	94	66.7	96.9
Klebsiella aerogenes	0	1	5	94	100.0	94.9
Ralstonia mannitolilytica	0	0	5	95	-	95.0
Prevotella oris	0	0	5	95	-	95.0
Bacteroides uniformis	0	0	5	95	-	95.0
Citrobacter koseri	0	0	5	95	-	95.0
Klebsiella quasipneumoniae	0	0	4	96	-	96.0
Neisseria flavescens	0	0	4	96	-	96.0
Methylorubrum extorquens	0	0	4	96	-	96.0
Burkholderia cenocepacia	0	0	3	97	-	97.0
Alloprevotella tannerae	0	0	3	97	-	97.0
Morganella morganii	0	1	1	98	100.0	99.0
Proteus vulgaris	1	0	1	98	0.0	99.0
Fusobacterium periodonticum	0	0	2	98	-	98.0
Porphyromonas gingivalis	0	0	2	98	-	98.0
Prevotella salivae	0	0	2	98	-	98.0
Veillonella dispar	0	0	2	98	-	98.0
Bacteroides fragilis	0	0	2	98	-	98.0
Campylobacter rectus	0	0	2	98	-	98.0
Chryseobacterium indologenes	0	0	2	98	-	98.0
Citrobacter youngae	0	0	2	98	-	98.0
Delftia tsuruhatensis	0	0	2	98	-	98.0
Haemophilus influenzae	0	0	2	98	-	98.0
Mycoplasma orale	0	0	2	98	-	98.0
Prevotella pallens	0	0	2	98	-	98.0
Proteus mirabilis	0	0	2	98	-	98.0

Tannerella forsythia	0	0	2	98	-	98.0
Bradyrhizobium elkanii	0	0	2	98	-	98.0
Veillonella atypica	0	0	2	98	-	98.0
Achromobacter xylosoxidans	0	1	0	99	100.0	100.0
Serratia ureilytica	1	0	0	99	0.0	100.0
Achromobacter insuavis	0	0	1	99	-	99.0
Alloprevotella rava	0	0	1	99	-	99.0
Bifidobacterium dentium	0	0	1	99	-	99.0
Burkholderia ubonensis	0	0	1	99	-	99.0
Delftia acidovorans	0	0	1	99	-	99.0
Elizabethkingia anophelis	0	0	1	99	-	99.0
Haemophilus parahaemolyticus	0	0	1	99	-	99.0
Klebsiella variicola	0	0	1	99	-	99.0
Megasphaera micronuciformis	0	0	1	99	-	99.0
Neisseria mucosa	0	0	1	99	-	99.0
Neisseria sicca	0	0	1	99	-	99.0
Porphyromonas endodontalis	0	0	1	99	-	99.0
Selenomonas flueggei	0	0	1	99	-	99.0
Acinetobacter pittii	0	0	1	99	-	99.0
Arcobacter cryaerophilus	0	0	1	99	-	99.0
Elizabethkingia meningoseptica	0	0	1	99	-	99.0
Helicobacter pylori	0	0	1	99	-	99.0
Methyloversatilis discipulorum	0	0	1	99	-	99.0
Prevotella copri	0	0	1	99	-	99.0
Prevotella intermedia	0	0	1	99	-	99.0
Selenomonas noxia	0	0	1	99	-	99.0
Sphingomonas echinoides	0	0	1	99	-	99.0
**Bacteria, Gram-positive**	6	4	145	5245	40.0	97.3
Cutibacterium acnes	0	0	14	86	-	86.0
Staphylococcus aureus	3	3	7	87	50.0	92.6
Staphylococcus epidermidis	1	0	10	89	0.0	89.9
Enterococcus faecium	0	0	10	90	-	90.0
Streptococcus anginosus	0	1	6	93	100.0	93.9
Enterococcus faecalis	0	0	7	93	-	93.0
Staphylococcus haemolyticus	0	0	6	94	-	94.0
Streptococcus mitis	0	0	6	94	-	94.0
Staphylococcus hominis	0	0	6	94	-	94.0
Corynebacterium striatum	0	0	5	95	-	95.0
Propionibacterium humerusii	0	0	5	95	-	95.0
Staphylococcus warneri	0	0	4	96	-	96.0
Corynebacterium resistens	0	0	3	97	-	97.0
Lactobacillus fermentum	0	0	3	97	-	97.0
Streptococcus constellatus	0	0	3	97	-	97.0
Streptococcus oralis	0	0	3	97	-	97.0
Corynebacterium accolens	0	0	3	97	-	97.0
Staphylococcus capitis	1	0	1	98	0.0	99.0
Streptococcus parasanguinis	0	0	2	98	-	98.0
Enterococcus hirae	0	0	2	98	-	98.0

Lactobacillus mucosae	0	0	2	98	-	98.0
Mycobacterium lentiflavum	0	0	2	98	-	98.0
Staphylococcus argenteus	0	0	2	98	-	98.0
Streptococcus agalactiae	0	0	2	98	-	98.0
Corynebacterium matruchotii	0	0	2	98	-	98.0
Streptococcus pneumoniae	0	0	2	98	-	98.0
Corynebacterium species	1	0	0	99	0.0	100.0
Actinomyces graevenitzii	0	0	1	99	-	99.0
Atopobium parvulum	0	0	1	99	-	99.0
Corynebacterium callunae	0	0	1	99	-	99.0
Corynebacterium jeikeium	0	0	1	99	-	99.0
Corynebacterium propinquum	0	0	1	99	-	99.0
Corynebacterium pseudogenitalium	0	0	1	99	-	99.0
Eggerthia catenaformis	0	0	1	99	-	99.0
Enterococcus avium	0	0	1	99	-	99.0
Enterococcus raffinosus	0	0	1	99	-	99.0
Filifactor alocis	0	0	1	99	-	99.0
Lactobacillus crispatus	0	0	1	99	-	99.0
Lactobacillus gasseri	0	0	1	99	-	99.0
Lactobacillus paracasei	0	0	1	99	-	99.0
Lactobacillus salivarius	0	0	1	99	-	99.0
Parvimonas micra	0	0	1	99	-	99.0
Sanguibacter keddieii	0	0	1	99	-	99.0
Schaalia odontolytica	0	0	1	99	-	99.0
Streptococcus mutans	0	0	1	99	-	99.0
Streptococcus salivarius	0	0	1	99	-	99.0
Tropheryma whipplei	0	0	1	99	-	99.0
Weissella confusa	0	0	1	99	-	99.0
Anaerococcus prevotii	0	0	1	99	-	99.0
Bifidobacterium thermophilum	0	0	1	99	-	99.0
Corynebacterium aurimucosum	0	0	1	99	-	99.0
Streptococcus intermedius	0	0	1	99	-	99.0
Streptococcus pseudopneumoniae	0	0	1	99	-	99.0
Streptococcus sanguinis	0	0	1	99	-	99.0
**Fungus**	14	10	28	1448	41.7	98.1
Candida albicans	5	9	6	80	64.3	93.0
Candida glabrata	3	0	2	95	0.0	97.9
Candida tropicalis	3	0	2	95	0.0	97.9
Candida parapsilosis	1	0	4	95	0.0	96.0
Aspergillus turcosus	0	0	4	96	-	96.0
Pneumocystis jirovecii	0	0	3	97	-	97.0
Cladosporium sphaerospermum	0	0	2	98	-	98.0
Candida haemulonii	1	0	0	99	0.0	100.0
Cryptococcus neoformans_var_grubii	1	0	0	99	0.0	100.0
Trichosporon asahii	0	1	0	99	100.0	100.0
Malassezia restricta	0	0	1	99	-	99.0

Schizophyllum commune	0	0	1	99	-	99.0
Aureobasidium pullulans	0	0	1	99	-	99.0
Aspergillus versicolor	0	0	1	99	-	99.0
Melampsora pinitorqua	0	0	1	99	-	99.0
**Virus**	0	3	71	1426	100.0	95.3
CMV	0	2	20	78	100.0	79.6
EBV	0	0	17	83	-	83.0
HSV1	0	1	12	87	100.0	87.9
Human polyomavirus 1	0	0	3	97	-	97.0
Human beta-herpesvirus 6B	0	0	3	97	-	97.0
JC polyomavirus	0	0	3	97	-	97.0
Human beta-herpesvirus 7	0	0	2	98	-	98.0
Human gamma-herpesvirus 8	0	0	2	98	-	98.0
Trichodysplasia spinulosa-associated polyomavirus	0	0	2	98	-	98.0
VZV	0	0	2	98	-	98.0
BK polyomavirus1	0	0	1	99	-	99.0
HPV16	0	0	1	99	-	99.0
Hepatitis B virus	0	0	1	99	-	99.0
Human alphaherpesvirus 2	0	0	1	99	-	99.0
Human RSVA	0	0	1	99	-	99.0
**Mycobacteria**	4	0	0	296	0.0	100.0
Mycobacterium avium	2	0	0	98	0.0	100.0
Mycobacterium mantenii	1	0	0	99	0.0	100.0
Mycobacterium tuberculosis	1	0	0	99	0.0	100.0
**Other**						
Toxoplasma gondii	0	0	1	99	-	99.0
**All**	30	49	499	15222	62.0	96.8

mNGS, metagenomic next-generation sequencing; NPA, negative percent agreement; PPA, positive percentage agreement; BK virus, human polyomavirus; CMV, cytomegalovirus; EBV, Epstein-Barr virus; HPV16, human papillomavirus type 16; HSV, Herpes simplex virus; RSVA, respiratory syncytial virus subtype A; VZV, varicella-zoster virus.

Supplementary TABLE 4 Distribution and classification of reads among specimens

	Blood			Bronchial alveolar lavage fluid					
	DNA			DNA			RNA		
Case No.	Raw reads	Human reads (%)	Microbial reads	Raw reads	Human reads (%)	Microbial reads	Raw reads	Human reads (%)	Microbial reads
1	113,553,772	113,526,476 (99.46%)	27,296	52,559,493	52,543,278 (99.41%)	16,215	27,726,591	27,717,209 (98.64%)	9,382
2	116,221,278	116,187,176 (99.44%)	34,102	59,009,230	58,969,467 (99.29%)	39,763	52,302,031	52,248,248 (98.50%)	53,783
3	90,351,722	90,314,272 (99.41%)	37,450	35,579,134	35,504,302 (99.06%)	74,832	40,192,361	40,081,436 (98.14%)	110,925
4	150,936,307	150,896,638 (99.46%)	39,669	44,201,769	44,169,614 (99.34%)	32,155	28,078,070	28,050,090 (96.92%)	27,980
5	116,458,889	116,419,558 (99.41%)	39,331	40,164,886	40,130,783 (99.20%)	34,103	39,391,984	39,365,551 (98.33%)	26,433
6	114,815,339	114,741,138 (99.25%)	74,201	52,727,010	52,686,302 (99.21%)	40,708	46,474,245	46,437,612 (98.29%)	36,633
7	126,968,485	126,910,327 (99.31%)	58,158	33,152,980	33,096,317 (99.17%)	56,663	31,368,186	31,277,867 (97.89%)	90,319
8	103,474,830	103,442,110 (99.37%)	32,720	47,218,250	47,193,413 (99.32%)	24,837	45,060,608	45,033,673 (98.55%)	26,935
9	67,263,008	67,227,752 (99.17%)	35,256	55,413,357	55,337,958 (99.02%)	75,399	27,353,535	27,308,979 (97.77%)	44,556
10	153,468,226	153,401,720 (99.15%)	66,506	51,148,585	51,110,445 (99.21%)	38,140	75,290,504	75,258,122 (98.59%)	32,382
11	126,793,613	126,748,376 (99.20%)	45,237	34,020,633	34,006,565 (99.42%)	14,068	84,063,920	84,022,617 (96.36%)	41,303
12	103,988,751	103,931,956 (99.16%)	56,795	29,435,832	29,359,958 (99.06%)	75,874	54,034,600	53,675,229 (97.62%)	359,371
13	81,067,962	81,032,033 (99.29%)	35,929	42,579,479	42,471,693 (98.33%)	107,786	34,769,995	34,678,375 (86.20%)	91,620
14	119,750,487	119,652,362 (99.29%)	98,125	31,412,966	31,330,998 (99.10%)	81,968	20,559,128	20,470,007 (97.43%)	89,121
15	105,455,509	105,369,791 (99.21%)	85,718	23,244,962	23,124,983 (98.80%)	119,979		Not available	
17	166,214,926	166,146,103 (99.28%)	68,823	29,797,020	29,770,872 (99.20%)	26,148	73,858,289	73,827,597 (98.90%)	30,692
18	139,180,414	139,100,342 (99.01%)	80,072	36,588,948	36,522,629 (97.41%)	66,319	28,472,263	28,455,816 (97.58%)	16,447
19	115,072,652	115,039,205 (99.17%)	33,447	43,839,795	43,734,200 (96.99%)	105,595	53,616,969	53,547,290 (97.65%)	69,679
20	125,585,030	125,436,256 (98.73%)	148,774	67,773,036	67,728,636 (98.53%)	44,400	26,877,777	26,860,577 (97.59%)	17,200
21	140,304,737	140,238,596 (99.05%)	66,141	26,175,112	26,165,153 (98.44%)	9,959		Not available	
22	157,674,176	157,512,697 (98.87%)	161,479	54,178,159	54,149,584 (98.60%)	28,575	42,996,692	42,970,316 (97.60%)	26,376
23	107,830,304	107,748,091 (98.99%)	82,213	30,957,076	30,899,909 (98.00%)	57,167	52,333,883	52,240,328 (97.15%)	93,555
24	103,563,052	103,478,342 (99.02%)	84,710	37,054,789	37,000,406 (97.73%)	54,383	32,752,931	32,730,676 (97.20%)	22,255
25	67,016,480	66,979,035 (99.15%)	37,445	37,552,938	37,532,705 (98.77%)	20,233	23,735,158	23,723,289 (97.52%)	11,869
26	118,473,455	118,408,580 (98.93%)	64,875	51,215,576	51,187,566 (98.49%)	28,010		Not available	
27	173,318,002	173,243,523 (99.02%)	74,479	37,122,762	37,001,733 (97.00%)	121,029	59,318,020	59,133,980 (96.48%)	184,040
28	59,599,758	59,551,395 (98.83%)	48,363	33,729,092	33,647,921 (96.76%)	81,171	89,672,449	89,631,855 (97.53%)	40,594
29	100,580,683	100,399,102 (98.37%)	181,581	45,421,998	45,285,106 (97.03%)	136,892	33,080,378	32,986,368 (95.55%)	94,010
30	131,605,337	131,463,732 (98.71%)	141,605	73,257,409	73,200,024 (98.11%)	57,385		Not available	
31	42,277,909	42,238,698 (98.67%)	39,211	30,761,942	30,741,092 (98.38%)	20,850	41,949,092	41,918,574 (96.86%)	30,518
32	152,935,245	152,868,639 (98.91%)	66,606	45,618,033	45,502,435 (97.89%)	115,598	33,102,668	32,799,182 (95.44%)	303,486
33	87,174,482	87,113,553 (98.87%)	60,929	24,492,310	24,454,466 (97.84%)	37,844		Not available	
34	154,018,070	153,934,196 (98.99%)	83,874	18,056,136	17,932,494 (97.85%)	123,642		Not available	
36	138,016,849	137,942,194 (99.06%)	74,655	21,840,555	21,815,891 (98.90%)	24,664	16,930,997	16,825,307 (97.61%)	105,690
37	91,485,309	91,330,283 (98.43%)	155,026		Not available		59,960,473	59,704,365 (90.39%)	256,108
38	179,900,923	179,788,585 (98.97%)	112,338	19,200,917	19,190,925 (99.05%)	9,992	43,431,166	43,406,639 (98.49%)	24,527
39	108,856,080	108,745,610 (98.65%)	110,470	29,660,839	29,635,505 (98.77%)	25,334	59,574,465	59,535,554 (98.53%)	38,911
40	119,986,782	119,916,275 (98.85%)	70,507	65,301,936	65,251,853 (98.84%)	50,083	32,764,722	32,731,456 (98.41%)	33,266
41	117,213,122	117,151,609 (98.95%)	61,513	56,359,386	56,283,201 (94.77%)	76,185	66,191,115	66,115,610 (98.11%)	75,505
42	114,449,089	114,383,712 (98.83%)	65,377	60,813,148	60,769,959 (99.03%)	43,189	31,326,956	31,302,783 (97.80%)	24,173
43	143,440,309	143,348,059 (98.72%)	92,250	69,396,630	69,332,098 (98.91%)	64,532	26,100,329	26,060,255 (97.92%)	40,074
44	100,383,692	100,284,070 (98.77%)	99,622	56,061,415	55,995,729 (98.83%)	65,686	48,357,851	48,204,108 (97.91%)	153,743
45	86,502,046	86,458,683 (98.99%)	43,363	44,150,323	44,062,621 (97.39%)	87,702	48,626,764	48,556,369 (96.36%)	70,395
46	80,498,829	80,441,693 (99.04%)	57,136	33,352,394	33,326,574 (98.97%)	25,820	12,321,185	12,310,997 (92.99%)	10,188
47	63,017,546	62,979,600 (99.20%)	37,946	57,273,896	57,204,968 (98.91%)	68,928	24,502,079	24,476,464 (97.30%)	25,615
48	110,671,010	110,618,211 (99.30%)	52,799	61,058,668	61,016,212 (98.93%)	42,456	31,361,259	31,334,889 (97.23%)	26,370
49	112,748,162	112,674,411 (99.10%)	73,751	35,679,323	35,580,611 (96.95%)	98,712	32,912,198	32,751,472 (94.52%)	160,726
50	49,144,969	49,112,567 (99.15%)	32,402	62,861,332	62,799,404 (98.63%)	61,928	35,431,116	35,381,895 (97.45%)	49,221
51	139,616,347	139,549,424 (99.08%)	66,923	71,114,748	71,075,366 (99.09%)	39,382	33,229,835	33,186,644 (96.56%)	43,191
52	156,093,080	156,040,311 (99.35%)	52,769	63,084,712	63,053,215 (99.10%)	31,497	51,892,054	51,731,846 (93.14%)	160,208
